# Causal relationship between the gut microbiota and insomnia: a two-sample Mendelian randomization study

**DOI:** 10.3389/fcimb.2024.1279218

**Published:** 2024-03-04

**Authors:** Qianfei Wang, Tianci Gao, Weichao Zhang, Dong Liu, Xin Li, Fenqiao Chen, Jianqiang Mei

**Affiliations:** ^1^ The Graduate School, Hebei University of Chinese Medicine, Shijiazhuang, China; ^2^ The First Affiliated Hospital, Hebei University of Chinese Medicine, Shijiazhuang, China

**Keywords:** insomnia, gut microbiota, causal relationship, Mendelian randomization, bacterial traits

## Abstract

**Background:**

Changes in the gut microbiota are closely related to insomnia, but the causal relationship between them is not yet clear.

**Objective:**

To clarify the relationship between the gut microbiota and insomnia and provide genetic evidence for them, we conducted a two-sample Mendelian randomization study.

**Methods:**

We used a Mendelian randomized two-way validation method to discuss the causal relationship. First, we downloaded the data of 462,341 participants relating to insomnia, and the data of 18,340 participants relating to the gut microbiota from a genome-wide association study (GWAS). Then, we used two regression models, inverse-variance weighted (IVW) and MR-Egger regression, to evaluate the relationship between exposure factors and outcomes. Finally, we took a reverse MR analysis to assess the possibility of reverse causality.

**Results:**

The combined results show 19 gut microbiotas to have a causal relationship with insomnia (odds ratio (OR): 1.03; 95% confidence interval (CI): 1.01, 1.05; p=0.000 for class. Negativicutes; OR: 1.03; 95% CI: 1.01, 1.05; p=0.000 for order.Selenomonadales; OR: 1.01; 95% CI: 1.00, 1.02; p=0.003 for genus.RikenellaceaeRC9gutgroup). The results were consistent with sensitivity analyses for these bacterial traits. In reverse MR analysis, we found no statistical difference between insomnia and these gut microbiotas.

**Conclusion:**

This study can provide a new direction for the causal relationship between the gut microbiota (class.Negativicutes, order.Selenomonadales, genus.Lactococcus) and insomnia and the treatment or prevention strategies of insomnia.

## 1 Introduction

Insomnia refers to a sleep disorder characterized by frequent and persistent difficulty falling asleep or maintaining sleep despite appropriate sleep opportunities and sleep environments (Sutton, 2021; Cunnington et al., 2013). The disease is mainly characterized by difficulty in falling asleep, dreaminess, easy awakening, and early awakening and is often accompanied by physical symptoms (pain, nervous, and numbness) and mental disorders (depression, anxiety, and irritability). According to the statistics, more than 30% of the worldwide population experiences one or more symptoms of insomnia (Madari et al., 2021), seriously affecting the lives and work of patients. The pathogenesis of insomnia is very complicated. The occurrence and development of insomnia are closely related to individual factors and various environmental factors. Susceptible factors, inducing factors, and maintaining factors play a very important role (Proserpio et al., 2020).

Recently, increasing evidence shows that changes in the gut microbiota are closely related to host health (Agus et al., 2018; Morrison and Preston, 2016). The microbiota–gut–brain axis has been confirmed to be related to multi-system diseases, such as the nervous system, and participates in the pathogenesis of many mental diseases (Forslund et al., 2017; Cox and Weiner, 2018; Loo et al., 2020). The gut microbiota is called the “second genome” of the human body (Preethy et al., 2022). The ratio of bacteria to human cells is now considered to be closer to 1:1, and the genes it contains are 100 times that of human coding genes. The gut microbiota has been shown to regulate body health and brain function by participating in food digestion and decomposition (Burokas et al., 2017), regulating bile acid metabolism (Burokas et al., 2017), resisting pathogen invasion (Cheng et al., 2019), and participating in immune response (Yang and Cong, 2021). At present, there have been reports about insomnia and the gut microbiota. Thaiss et al. (2016) found that, on the one hand, changing the sleep pattern of mice can change the structure and diversity of their gut microbiota, and on the other hand, changes in the gut microbiota can affect the diurnal fluctuations of host physiology and disease susceptibility. Hua et al. (2020) found that the abundance of some bacteria in autistic children with sleep disorders decreased, which further affected the changes in levels of various neurotransmitters, and which, in turn, may aggravate sleep problems and autism spectrum disorder in children. However, the specific mechanism has not been reported. Therefore, exploring the causal relationship between the gut microbiota and insomnia is particularly important for explaining the incidence, treatment, and prevention of insomnia.

Mendelian randomization study (MR) is a method used in epidemiology to evaluate the relationship between disease exposure factors and disease risk (Sekula et al., 2016; Chen et al., 2021). The genetic variation is used as an instrumental variable to control the bias of causality estimation between disease and exposure factors and eliminate the influence of unmeasured confounders so as to draw strong causal inference. It has been used in the study of many diseases, such as those investigating the relationship between coffee consumption and kidney function (Kennedy et al., 2020), smoking and stroke (Larsson et al., 2019), and rheumatoid arthritis and osteoporosis (Liu et al., 2021). This study is based on pooled data from the Genome-Wide Association Study (GWAS) and explores the causal relationship between the gut microbiota and insomnia using a two-sample MR method.

## 2 Materials and methods

### 2.1 Study design process

**Figure 1:**
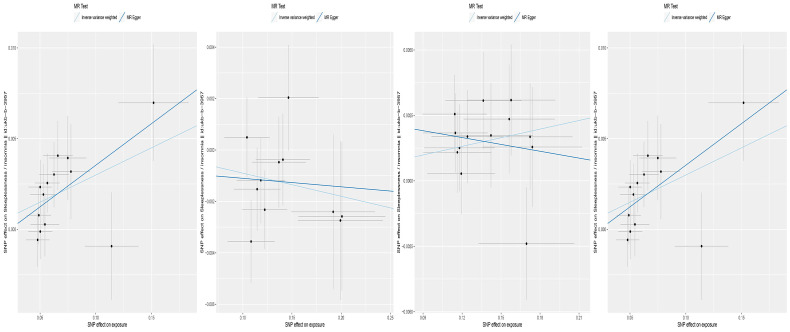
The study design of the associations between gut microbiota and insomnia.

The exposure data and outcome data in this article are derived from public databases, so this study does not require ethical approval, an informed consent form, or other relevant materials. We used the MR method to analyze the causal relationship between gut microbiota and insomnia using genetic instrumental variables (IVs). The study flow chart is shown in **Figure 1**.

### 2.2 Source of exposure factors

We downloaded the gut microbiota data from https://mibiogen.gcc.rug.nl/, the largest, multi-ethnic genome-wide meta-analysis of gut microbes to date. The data is a collection of 16S rRNA gene sequencing profiles and genotyping data from 18,340 subjects from 11 countries in Asia and Europe by the MiBioGen Consortium. It maps the microbiome signature gene to identify genetic loci that influence the relative abundance or presence of microbial taxa, all of which are in the GWAS database. We diluted all datasets to 10,000 reads per sample to account for differences in sequencing depth and to classify them. The correlation analysis of age, sex, technical covariates, and genetic principal components was carried out. Finally, after eliminating unknown gut microbiotas, all available GWAS summary statistics of 196 bacterial taxa (including 9 Phylums, 16 Classes, 20 Orders, 32 Families, and 119 Genera) were eventually included in the MR analysis.

### 2.3 Source of outcome factors

We obtained online insomnia data through GWAS (https://gwas.mrcieu.ac.uk/), Dataset: ukb-b-3957, POPULATION: European, sex: male and female, samples size: 462,341, number of SNPs: 9,851,867.

### 2.4 Selection of IVs

We screened for the statistically significant single-nucleotide polymorphisms (SNPs) as IVs for subsequent studies. After extracting the relevant information, we used the formula [R^2^=2×MAF× (1−MAF) ×β^2^, F= R^2^ (n-k-1)/k (1-R^2^), MAF: minor allele frequency of SNPs, n: the sample size, k: the number of IVs employed] to calculate the proportion of variation explained (R^2^) and F-statistics. We defined the condition of IVs, which is p < 1 × 10^−5^, the linkage disequilibrium (LD) threshold at R^2^<0.001, and clumping distance = 10,000 kb to ensure IVs were independent of each other (Ning et al., 2022; Johnson et al., 2008). The SNPs with the lowest p-value associated traits were retained and 196 bacterial traits were clustered. Then, with the help of the screened SNPs, the relationship between the gut microbiota and insomnia was identified using different algorithms including the inverse-variance weighted (IVW) method, weighted median method, MR-Egger regression, and the MR pleiotropy residual sum and outlier (MR-PRESSO) test. Finally, IVs were set to (p<1×10^−8^) in reverse MR analysis.

### 2.5 Main analysis

We analyzed the relationship between the gut microbiota and insomnia using the IVW method, which provides the most accurate effect estimation for this study. Therefore, the IVW method was used as the main analysis method in this paper. When there is no pleiotropy in IV, the IVW method has high power and accuracy and can obtain the effect estimate with the minimum bias. In this paper, we obtained the IVW mean of SNP ratio estimates by regression of SNP-gut microbiota and SNP-insomnia association (Burgess et al., 2013).

### 2.6 Pleiotropy test

The IV pleiotropy increases the probability of type I errors in MR analysis, and if it is eliminated directly, it may lead to a weak IV (Burgess and Thompson, 2013). Therefore, we used MR-Egger regression to test for potential horizontal pleiotropy, and if P <0.05, horizontal pleiotropy was present. The MR-Egger regression method tests for potential horizontal pleiotropy, which is a supplementary method in this paper. MR-Egger regression, which weighted linear regression of SNP-gut microbiota and SNP-insomnia effect estimates, provided a valid causal effect estimate even when all SNPs were null instruments. When the direction of the two results is consistent, it is considered a relatively stable causal association.

### 2.7 Sensitivity analysis

Since the results of the IVW method are susceptible to valid instrumentation and potential pleiotropic effects, we performed sensitivity analyses to assess the robustness of the association, and when the results were consistent, it was indicated that the causal effect was significantly robust. This study also used the weighted median method for sensitivity analysis and comparison with the results obtained from the main analysis. Finally, we performed the MR-PRESSO test, conducting a global test of heterogeneity to identify whether SNPs were present with possible outliers, and we obtained corrected association results after the test.

### 2.8 Reverse MR analysis

Reverse MR analysis was performed only if all MR methods supported an association between the gut microbiota and insomnia. That is, the causal relationship between insomnia as exposure and the gut microbiota as outcome was assessed. The study sample, method principle, and analysis steps of the reverse MR analysis were the same as those of the forward MR analysis. The direction of the causal effect was further confirmed when the forward MR analysis was statistically significant and the reverse MR analysis was not.

### 2.9 Statistical analysis

The above analysis was completed using R 4.3.0 and RStudio software, and we conducted relevant research based on the “TwoSampleMR” and “ggplot2” package environments. The evaluation indexes were odds ratio (OR) and 95% confidence interval (95% CI), and P <0.05 was statistically significant.

## 3 Results

### 3.1 Main results of the 196 bacterial traits with the risk of insomnia

**Figure 2:**
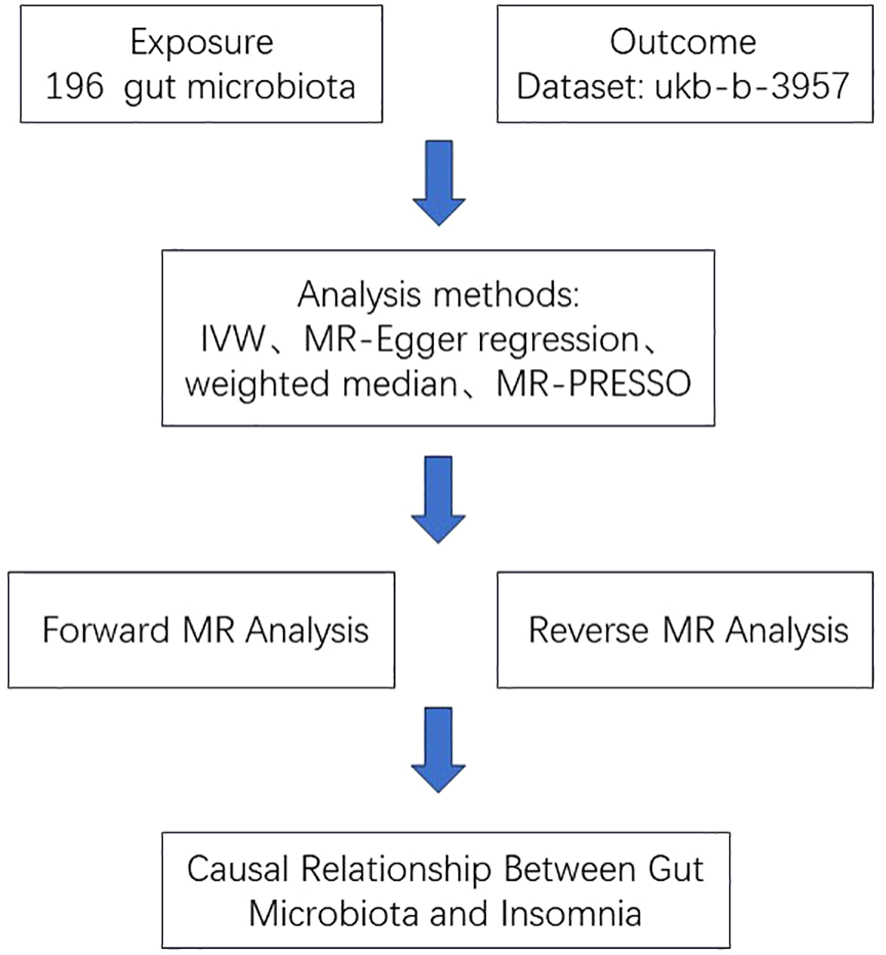
Forest plot of the associations between 19 genetically determined bacterial traits with the risk of insomnia.

We analyzed and identified 19 gut microbiotas that were significantly associated with insomnia using the IVW method (**Figure 2**).

**Figure 3:**
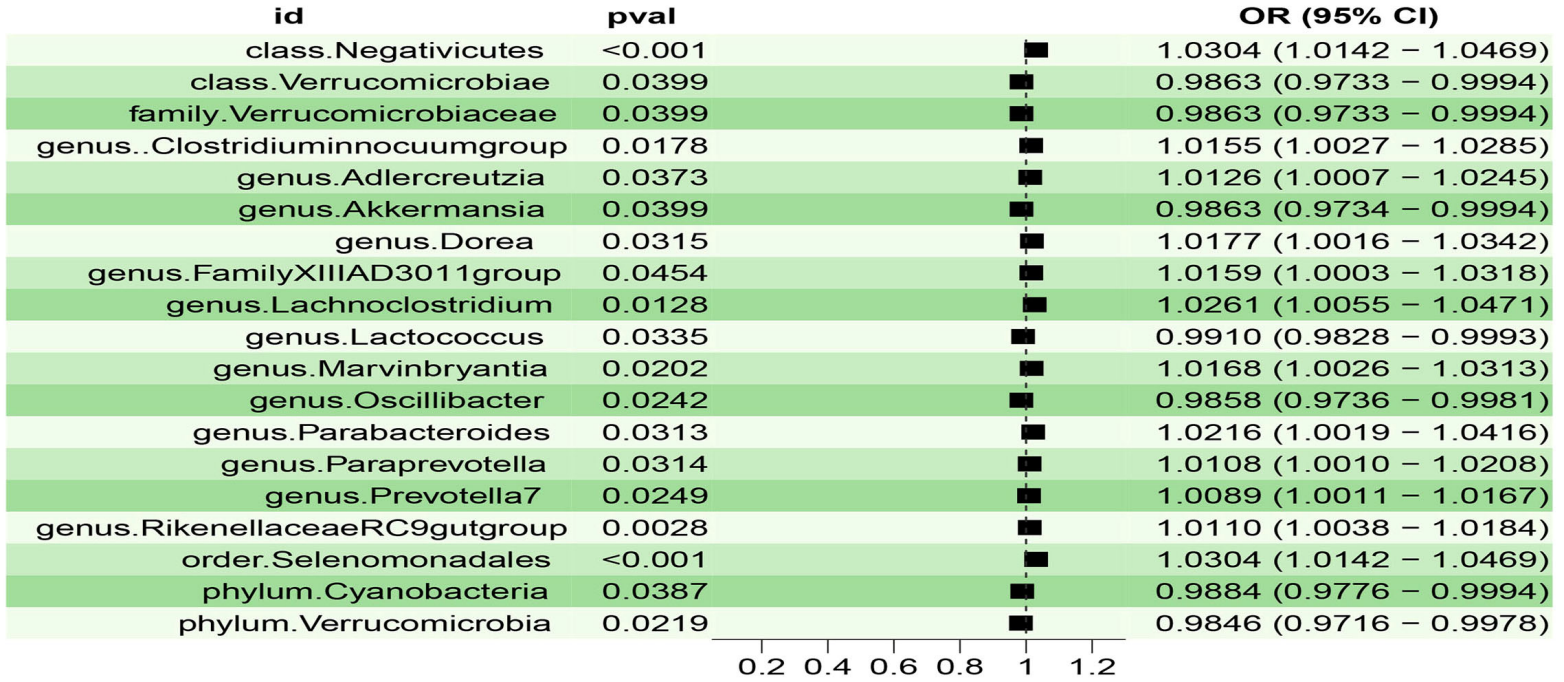
Scatter plot of the associations of genetic variants with four bacterial traits and the risk of Insomnia.

We found that the three gut microbiotas (class.Negativicutes, order.Selenomonadales, genus.RikenellaceaeRC9gutgroup) were significantly positively correlated with insomnia using the IVW method and the weighted-median method, whereas the MR-PRESSO test did not detect outliers (P<0.05), and MR-Egger regression showed no pleiotropy (P>0.05). One gut microbiota (genus.Lactococcus) was significantly negatively correlated with insomnia using the IVW method and the weighted-median method, whereas the MR-PRESSO test did not detect outliers (P <0.05), and MR-Egger regression showed no pleiotropy (P>0.05) (**Table 1**, **Figure 3**).

**Table 1:** Effect estimates of the associations of Insomnia with four gut microbiotas in the MR analysis.

Gut microbiome	Methods	N.SNPs	OR	95% CI	p-value	Intercept p-value
class.Negativicutes	Inverse-variance weighted	13	1.03	1.01-1.05	0.000	
	Weighted median	13	1.04	1.02-1.07	0.000	
	MR-PRESSO test	13	1.03	1.02-1.05	0.001	
	MR-Egger	13				0.105
order.Selenomonadales	Inverse-variance weighted	13	1.03	1.01-1.05	0.000	
	Weighted median	13	1.04	1.02-1.07	0.000	
	MR-PRESSO test	13	1.03	1.02-1.05	0.001	
	MR-Egger	13				0.105
genus.Lactococcus	Inverse-variance weighted	11	0.99	0.98-0.99	0.034	
	Weighted median	11	0.98	0.97-0.99	0.045	
	MR-PRESSO test	11	0.99	0.98-0.99	0.033	
	MR-Egger	11				0.879
genus.RikenellaceaeRC9gutgroup	Inverse-variance weighted	13	1.01	1.00-1.02	0.003	
	Weighted median	13	1.01	1.00-1.02	0.001	
	MR-PRESSO test	13	1.01	1.00-1.01	0.001	
	MR-Egger	13				0.714

We found that the other 15 gut microbiotas were significantly associated with insomnia using the IVM method, (OR: 0.98; 95% CI: 0.97, 0.99; p=0.039, for class.Verrucomicrobiae; OR: 0.98; 95% CI: 0.97, 0.99; p=0.039, for family.Verrucomicrobiaceae; OR: 1.01; 95% CI: 1.00, 1.02; p=0.017, for genus.Clostridiuminnocuumgroup; OR: 1.01; 95% CI: 1.00, 1.02; p=0.037, for genus.Adlercreutzia; OR: 0.98; 95% CI: 0.97, 0.99; p=0.039, for genus.Akkermansia; OR: 1.01; 95% CI: 1.00, 1.03; p=0.031, for genus.Dorea; OR: 1.01; 95% CI: 1.00, 1.03; p=0.045, for genus.FamilyXIIIAD3011group; OR: 1.02; 95% CI: 1.00, 1.04; p=0.012, for genus.Lachnoclostridium; OR: 1.01; 95% CI: 1.00, 1.03; p=0.020, for genus.Marvinbryantia; OR: 0.98; 95% CI: 0.97, 0.99; p=0.024, for genus.Oscillibacter; OR: 1.02; 95% CI: 1.00, 1.04; p=0.031, for genus.Parabacteroides; OR: 1.01; 95% CI: 1.00, 1.02; p=0.031, for genus.Paraprevotella; OR: 1.00; 95% CI: 1.00, 1.01; p=0.024, for genus.Prevotella7; OR: 0.98; 95% CI: 0.97, 0.99; p=0.038, for phylum.Cyanobacteria; OR: 0.98; 95% CI: 0.97, 0.99; p=0.021, for phylum.Verrucomicrobia); however, this causal relationship was not supported (P>0.05) in the weighted median method results.

### 3.2 The result of reverse MR analysis

We performed reverse MR analysis of the four gut microbiotas. The results showed that we did not find a statistically significant association between Insomnia and any of the three gut microbiotas (class.Negativicutes, order.Selenomonadales, genus.Lactococcus) (**Table 2**). However, we found a statistically significant association between Insomnia and genus.Rikenellaceae RC9 gut group.

**Table 2:** Effect estimates of the associations of Insomnia with four gut microbiotas in the reverse MR analyses.

Gut microbiome	Methods	N.SNPs	OR	95% CI	p-value	Intercept p-value
class.Negativicutes	Inverse-variance weighted	32	1.37	0.94-1.99	0.099	
	Weighted median	32	1.42	0.84-2.38	0.189	
	MR-Egger	32				0.964
genus.Lactococcus	Inverse-variance weighted	31	0.63	0.26-1.54	0.313	
	Weighted median	31	0.70	0.22-2.29	0.558	
	MR-Egger	31				0.604
genus.RikenellaceaeRC9gutgroup	Inverse-variance weighted	30	0.39	0.15-0.99	0.046	
	Weighted median	30	0.30	0.08-1.18	0.085	
	MR-Egger	30				0.357
order.Selenomonadales	Inverse-variance weighted	32	1.37	0.94-1.99	0.099	
	Weighted median	32	1.42	0.85-2.34	0.182	
	MR-Egger	32				0.964

## 4 Discussion

Insomnia is a sleep disorder characterized by frequent and persistent difficulty falling asleep or maintaining sleep, resulting in inadequate sleep satisfaction. The pathogenesis of insomnia is complex, and it has been previously considered to be related to the abnormality of the GABAergic neuron system (Abad and Guilleminault, 2018), the dysfunction of the hypothalamic–pituitary–adrenal axis (HPA axis) (Dong et al., 2021), the dysfunction of melatonin and its receptor (Cardinali et al., 2012), and the imbalance of central neurotransmitter secretion. The gut microbiota plays an important role in digestion and metabolism and is a hot research topic. It can regulate the central nervous system through multiple neural pathways to affect the sleep and mental state of the host and participate in the occurrence of a variety of neuropsychiatric diseases. At present, many scholars have proposed that the gut microbiota is closely related to nervous system diseases. The gut microbiota can affect host behavior through the immune, neuroendocrine, and vagus pathways of the gut–brain axis, thus regulating the central nervous system bidirectionally (Socała et al., 2021; Góralczyk-Bińkowska et al., 2022; Agirman et al., 2021). Insomnia is an important nervous system disease. Many studies have reported that drug intervention can improve the metabolic abnormalities of enterobacter, enterococcus, brucella, and other bacteria and correct the phenomenon of intestinal flora disorder (Feng et al., 2023; Song et al., 2021; Wang et al., 2022). However, most of the previous studies involved the quantitative detection of intestinal flora, but no conclusion of causality between them was made.

To analyze the causal relationship between insomnia and the gut microbiota, we subsequently took a two-sample MR analysis using data from the GWAS database. The results showed that we identified 19 gut microbiotas associated with insomnia, for example, class.Verrucomicrobiae, family.Verrucomicrobiaceae, genus.Clostridiuminnocuumgroup. Among them, three gut microbiotas (class.Negativicutes, order.Selenomonadales, genus.Lactococcus) were analyzed by forward and reverse MR and various data analysis methods, indicating that there is strong evidence of a causal relationship between insomnia and that the three gut microbiotas are closely related to the risk of insomnia. However, genus.Rikenellaceae RC9 gut group and insomnia are mutually causal.

Class.Negativicutes is a gram-negative bacteria characterized by two cell membranes and is a common strictly anaerobic double-walled bacteria. Its potential to produce specific metabolites has not been fully studied. At present, class.Negativicutes is closely related to diseases such as COVID-19 (Song et al., 2023), low birthweight infants (Warner et al., 2016), and obesity (Hu et al., 2022). In this study, it was found that there was a significant causal relationship between class.Negativicutes and insomnia. Although there was no report on this exposure factor and outcome index in relevant studies and reports, we believed that it may play an important role in the occurrence and development of insomnia. In the future, we will focus on observing the changes of this flora in insomnia, so as to provide positive or negative proof for the causal relationship.

Order.Selenomonadales has also been identified as a risk factor for insomnia. Existing studies have shown that the bacteria play a role in Alzheimer’s disease (Zhuang et al., 2018), Idiopathic Nephrotic Syndrome (He et al., 2021), and autism spectrum disorder (Sun et al., 2019). Xia et al. showed that melatonin could effectively improve the changes of intestinal flora in the host, in which Selenomonadales was significantly reduced (Xia et al., 2022). We hypothesize that this order.Selenomonadales may play a crucial role in the progression of insomnia.

Genus.Lactococcus is a gram-positive bacterium of the family streptococcaceae of lactobacillus and is the only negatively correlated gut microbiota. In clinical microbiology laboratory specimens, it can be isolated from blood, urine, eye cultures, and more commonly from blood cultures of patients with endocarditis. A study has shown that the genus Lactococcus is closely related to insomnia in children (Nebbia et al., 2020).

This study has some advantages. Firstly, this study is based on the GWAS data with the largest sample size in the world, without animal and clinical trials, and is the most relevant study on the relationship between insomnia and the gut microbiota. Secondly, our identification of three gut microbiotas by MR analysis could provide evidence for subsequent studies and help provide new approaches to the targeted treatment of insomnia. Thirdly, the use of MR analysis to explore the association between insomnia and the gut microbiota can avoid the bias caused by reverse causality and unknown confounding, and the different MR methods give consistent causal effects, indicating the robustness of the results.

This study also has some limitations. Firstly, the population included in this study was European, and the lack of data on other ethnicities may have an impact on our causal relationship. Secondly, bacterial classification is only analyzed at the genus level, lacking more professional analysis such as species and strains. Thirdly, MR analysis of the two samples failed to group the two sexes and to understand whether there was a difference in virulence between the sexes.

## 5 Conclusion

The causal relationship between insomnia and the gut microbiota was evaluated by MR analysis.

Three gut microbiotas (class Verrucomicrobiae, family Verrucomicrobiaceae, genus Clostridium innocuum) play a key role in the progress of insomnia, which provides a new research direction for the pathogenesis and treatment of insomnia.

## Data availability statement

The original contributions presented in the study are included in the article/supplementary material. Further inquiries can be directed to the corresponding authors.

## Ethics statement

This article does not require ethical approval.

## Author contributions

QW: Data curation, Investigation, Software, Writing – original draft. TG: Writing – original draft. WZ: Writing – original draft. DL: Writing – original draft. XL: Writing – original draft. FC: Writing – review & editing. JM: Writing – review & editing.

## References

[B1] Abad V. C. Guilleminault C. (2018). Insomnia in elderly patients: recommendations for pharmacological management. Drugs Aging 35, 791–817. doi: 10.1007/s40266-018-0569-8. 30058034

[B2] Agirman G. Yu K. B. Hsiao E. Y. (2021). Signaling inflammation across the gut-brain axis. Science 374, 1087–1092. doi: 10.1126/science.abi6087. 34822299

[B3] Agus A. Planchais J. Sokol H. (2018). Gut microbiota regulation of tryptophan metabolism in health and disease. Cell Host Microbe 23, 716–724. doi: 10.1016/j.chom.2018.05.003. 29902437

[B4] Burgess S. Butterworth A. Thompson S. G. (2013). Mendelian randomization analysis with multiple genetic variants using summarized data. Genet. Epidemiol. 37, 658–665. doi: 10.1002/gepi.21758. 24114802 PMC4377079

[B5] Burgess S. Thompson S. G. (2013). Use of allele scores as instrumental variables for Mendelian randomization. Int. J. Epidemiol. 42, 1134–1144. doi: 10.1093/ije/dyt093. 24062299 PMC3780999

[B6] Burokas A. Arboleya S. Moloney R. D. Peterson V. L Murphy K. Clarke G.. (2017). Targeting the microbiota-gut-brain axis: prebiotics have anxiolytic and antidepressant-like effects and reverse the impact of chronic stress in mice. Biol. Psychiatry 82, 472–487. doi: 10.1016/j.biopsych.2016.12.031. 28242013

[B7] Cardinali D. P. Srinivasan V. Brzezinski A. Brown G. M. (2012). Melatonin and its analogs in insomnia and depression. J. Pineal Res. 52, 365–375. doi: 10.1111/j.1600-079X.2011.00962.x. 21951153

[B8] Chen X. Kong J. Pan J. Huang K. Zhou W. Diao X.. (2021). Kidney damage causally affects the brain cortical structure: A Mendelian randomization study. EBioMedicine 72, 103592. doi: 10.1016/j.ebiom.2021.103592. 34619639 PMC8498227

[B9] Cheng H. Y. Ning M. X. Chen D. K. Ma W. T. (2019). Interactions between the gut microbiota and the host innate immune response against pathogens. Front. Immunol. 10, 607. doi: 10.3389/fimmu.2019.00607. 30984184 PMC6449424

[B10] Cox L. M. Weiner H. L. (2018). Microbiota signaling pathways that influence neurologic disease. Neurotherapeutics 15, 135–145. doi: 10.1007/s13311-017-0598-8. 29340928 PMC5794708

[B11] Cunnington D. Junge M. F. Fernando A. T. (2013). Insomnia: prevalence, consequences and effective treatment. Med. J. Aust. 199, S36–S40. doi: 10.5694/mja13.10718 24138364

[B12] Dong Y. J. Jiang N. H. Zhan L. H. Teng X. Fang X. Lin M. Q.. (2021). Soporific effect of modified Suanzaoren Decoction on mice models of insomnia by regulating Orexin-A and HPA axis homeostasis. BioMed. Pharmacother. 143, 112141. doi: 10.1016/j.biopha.2021.112141. 34509822

[B13] Feng W. Yang Z. Liu Y. Chen R. Song Z. Pan G.. (2023). Gut microbiota: A new target of traditional Chinese medicine for insomnia. BioMed. Pharmacother. 160, 114344. doi: 10.1016/j.biopha.2023.114344. 36738504

[B14] Forslund K. Hildebrand F. Nielsen T. Falony G. Chatelier E. L. Sunagawa S.. (2017). Corrigendum: Disentangling type 2 diabetes and metformin treatment signatures in the human gut microbiota. Nature 545, 116. doi: 10.1038/nature22318. 28470190

[B15] Góralczyk-Bińkowska A. Szmajda-Krygier D. Kozłowska E. (2022). The microbiota-gut-brain axis in psychiatric disorders. Int. J. Mol. Sci. 23, 11245. doi: 10.3390/ijms231911245 36232548 PMC9570195

[B16] He H. Lin M. You L. Chen T. Liang Z. Li D.. (2021). Gut microbiota profile in adult patients with idiopathic nephrotic syndrome. BioMed. Res. Int. 2021, 8854969. doi: 10.1155/2021/8854969. 33681383 PMC7910048

[B17] Hu J. Guo P. Mao R. Ren Z. Wen J. Yang Q.. (2022). Gut microbiota signature of obese adults across different classifications. Diabetes Metab. Syndr. Obes. 15, 3933–3947. doi: 10.2147/DMSO.S387523. 36601354 PMC9807070

[B18] Hua X. Zhu J. Yang T. Guo M. Li Q. Chen J.. (2020). The gut microbiota and associated metabolites are altered in sleep disorder of children with autism spectrum disorders. Front. Psychiatry 11, 855. doi: 10.3389/fpsyt.2020.00855. 32982808 PMC7493623

[B19] Johnson A. D. Handsaker R. E. Pulit S. L. Nizzari M. M. O'Donnell C. J. de Bakker P. I. (2008). SNAP: a web-based tool for identification and annotation of proxy SNPs using HapMap. Bioinformatics 24, 2938–2939. doi: 10.1093/bioinformatics/btn564. 18974171 PMC2720775

[B20] Kennedy O. J. Pirastu N. Poole R. Fallowfield J. A. Hayes P. C. Grzeszkowiak E. J.. (2020). Coffee consumption and kidney function: A mendelian randomization study. Am. J. Kidney Dis. 75, 753–761. doi: 10.1053/j.ajkd.2019.08.025. 31837886

[B21] Larsson S. C. Burgess S. Michaëlsson K. (2019). Smoking and stroke: A mendelian randomization study. Ann. Neurol. 86, 468–471. doi: 10.1002/ana.25534. 31237718 PMC6701987

[B22] Liu Y. Q. Liu Y. Chen Z. Y. Li H. Xiao T. (2021). Rheumatoid arthritis and osteoporosis: a bi-directional Mendelian randomization study. Aging (Albany NY) 13, 14109–14130. doi: 10.18632/aging.v13i10. 34015765 PMC8202858

[B23] Loo Y. T. Howell K. Chan M. Zhang P. Ng K. (2020). Modulation of the human gut microbiota by phenolics and phenolic fiber-rich foods. Compr. Rev. Food Sci. Food Saf. 19, 1268–1298. doi: 10.1111/1541-4337.12563. 33337077

[B24] Madari S. Golebiowski R. Mansukhani M. P. Kolla B. P. (2021). Pharmacological management of insomnia. Neurotherapeutics 18, 44–52. doi: 10.1007/s13311-021-01010-z. 33527255 PMC8116439

[B25] Morrison D. J. Preston T. (2016). Formation of short chain fatty acids by the gut microbiota and their impact on human metabolism. Gut Microbes 7, 189–200. doi: 10.1080/19490976.2015.1134082. 26963409 PMC4939913

[B26] Nebbia S. Lamberti C. Lo Bianco G. Cirrincione S. Laroute V. Cocaign-Bousquet M.. (2020). Antimicrobial potential of food lactic acid bacteria: bioactive peptide decrypting from caseins and bacteriocin production. Microorganisms 9, 65. doi: 10.3390/microorganisms9010065. 33383704 PMC7824078

[B27] Ning J. Huang S. Y. Chen S. D. Zhang Y. R. Huang Y. Y. Yu J. T. (2022). Investigating casual associations among gut microbiota, metabolites, and neurodegenerative diseases: A mendelian randomization study. J. Alzheimers Dis. 87, 211–222. doi: 10.3233/JAD-215411. 35275534

[B28] Preethy S. Ranganathan N. Raghavan K. Dedeepiya V. D. Ikewaki N. Abraham S. J. K. (2022). Integrating the synergy of the gut microbiome into regenerative medicine: relevance to neurological disorders. J. Alzheimers Dis. 87, 1451–1460. doi: 10.3233/JAD-220313. 35466942 PMC9277691

[B29] Proserpio P. Marra S. Campana C. Agostoni E. C. Palagini L. Nobili L.. (2020). Insomnia and menopause: a narrative review on mechanisms and treatments. Climacteric 23, 539–549. doi: 10.1080/13697137.2020.1799973. 32880197

[B30] Sekula P. Del Greco M. F. Pattaro C. Köttgen A. (2016). Mendelian randomization as an approach to assess causality using observational data. J. Am. Soc. Nephrol. 27, 3253–3265. doi: 10.1681/ASN.2016010098. 27486138 PMC5084898

[B31] Socała K. Doboszewska U. Szopa A. Serefko A. Włodarczyk M. Zielińska A.. (2021). The role of microbiota-gut-brain axis in neuropsychiatric and neurological disorders. Pharmacol. Res. 172, 105840. doi: 10.1016/j.phrs.2021.105840. 34450312

[B32] Song J. Wu Y. Yin X. Ma H. Zhang J. (2023). The causal links between gut microbiota and COVID-19: A Mendelian randomization study. J. Med. Virol. 95, e28784. doi: 10.1002/jmv.28784. 37219044

[B33] Song Y. Shan B. Zeng S. Zhang J. Jin C. Liao Z.. (2021). Raw and wine processed Schisandra chinensis attenuate anxiety like behavior via modulating gut microbiota and lipid metabolism pathway. J. Ethnopharmacol 266, 113426. doi: 10.1016/j.jep.2020.113426. 33007392

[B34] Sun H. You Z. Jia L. Wang F. (2019). Autism spectrum disorder is associated with gut microbiota disorder in children. BMC Pediatr. 19, 516. doi: 10.1186/s12887-019-1896-6. 31881951 PMC6933684

[B35] Sutton E. L. (2021). Insomnia. Ann. Intern. Med. 174, ITC33–ITC48. doi: 10.7326/AITC202103160. 33683929

[B36] Thaiss C. A. Levy M. Korem T. Dohnalová L. Shapiro H. Jaitin D. A.. (2016). Microbiota diurnal rhythmicity programs host transcriptome oscillations. Cell 167, 1495–1510.e12. doi: 10.1016/j.cell.2016.11.003. 27912059

[B37] Wang Q. Chen B. Sheng D. Yang J. Fu S. Wang J.. (2022). Multiomics analysis reveals aberrant metabolism and immunity linked gut microbiota with insomnia. Microbiol. Spectr. 10, e0099822. doi: 10.1128/spectrum.00998-22. 36190400 PMC9602994

[B38] Warner B. B. Deych E. Zhou Y. Hall-Moore C. Weinstock G. M. Sodergren E.. (2016). Gut bacteria dysbiosis and necrotising enterocolitis in very low birthweight infants: a prospective case-control study. Lancet 387, 1928–1936. doi: 10.1016/S0140-6736(16)00081-7. 26969089 PMC5553277

[B39] Xia S. Gao W. Li Y. Ma J. Gong S. Gao Z.. (2022). Effects of melatonin on intestinal function and bacterial compositions in sucking piglets [published correction appears in J Anim Physiol Anim Nutr (Berl). 2023 May;107(3):980]. J. Anim. Physiol. Anim. Nutr. (Berl) 106, 1139–1148. doi: 10.1111/jpn.13675. 35023236

[B40] Yang W. Cong Y. (2021). Gut microbiota-derived metabolites in the regulation of host immune responses and immune-related inflammatory diseases. Cell Mol. Immunol. 18, 866–877. doi: 10.1038/s41423-021-00661-4. 33707689 PMC8115644

[B41] Zhuang Z. Q. Shen L. L. Li W. W. Fu X. Zeng F. Gui L.. (2018). Gut microbiota is altered in patients with alzheimer's disease. J. Alzheimers Dis. 63, 1337–1346. doi: 10.3233/JAD-180176. 29758946

